# Exercício Aeróbio e Função Cardíaca de Murinos Expostos à Doxorrubicina: uma Metanálise

**DOI:** 10.36660/abc.20190260

**Published:** 2020-11-01

**Authors:** Mariana Inocêncio Matos, Ercole da Cruz Rubini, Frederico de Oliveira Meirelles, Elirez Bezerra da Silva

**Affiliations:** 1 Universidade do Estado do Rio de Janeiro Rio de JaneiroRJ Brasil Universidade do Estado do Rio de Janeiro - Programa de Pós-Graduação em Ciências do Exercício e do Esporte, Rio de Janeiro, RJ - Brasil; 2 Universidade do Estado do Rio de Janeiro Rio de JaneiroRJ Brasil Universidade do Estado do Rio de Janeiro - Grupo de Pesquisa em Ciências do Exercício e da Saúde, Rio de Janeiro, RJ - Brasil; 3 Universidade Estácio de Sá Rio de JaneiroRJ Brasil Universidade Estácio de Sá, Rio de Janeiro, RJ – Brasil

**Keywords:** Muridae, Exercício Físico, Antibacterianos, Doxorrubicina, Metanálise

## Abstract

**Fundamento:**

A cardiotoxicidade pode ser uma consequência do tratamento com doxorrubicina (DOX).

**Objetivos:**

Verificar o efeito do exercício aeróbio na prevenção da disfunção cardíaca de murinos expostos à DOX.

**Método:**

Uma busca abrangente foi realizada em nove bases de dados, em dezembro de 2017. Estudos que avaliaram a função cardíaca de murinos expostos à DOX foram incluídos. O nível de significância adotado foi de 5%.

**Resultados:**

Na comparação entre 230 murinos submetidos a exercício aeróbio mais DOX e 222 controles (tratados somente com DOX), a fração de encurtamento mostrou uma melhora de 5,33% a favor do grupo experimental (p = 0,0001). A pressão desenvolvida no ventrículo esquerdo também mostrou um aumento de 24,84 mmHg a favor do grupo de 153 murinos que realizaram exercício em comparação com o grupo controle de 166 murinos (p = 0,00001).

**Conclusão:**

Estudos pré-clínicos incluídos nesta metanálise indicaram que o exercício é uma boa estratégia não farmacológica para preservar a função cardíaca pós-DOX.

## Introdução

A quimioterapia expõe um novo panorama na oncologia, no qual a sobrevida dos pacientes tem aumentado junto com a vulnerabilidade à cardiotoxicidade adquirida em tratamentos avançados.^1^ Os efeitos da toxicidade gerada pelos agentes antineoplásicos usados no tratamento podem se manifestar imediatamente, durante a administração ou até anos depois.^2^^,^^3^ Entre os órgãos afetados, o coração merece atenção especial, já que a insuficiência cardíaca, muitas vezes adquirida após o tratamento químico, tem prognóstico igual ou pior quando comparada a cânceres localizados no fígado, intestino, bexiga, próstata, mama e ovário. Assim, tais complicações podem interromper o tratamento e comprometer a probabilidade de cura.^4^

A doxorrubicina (DOX) é um agente quimioterápico eficiente na luta contra câncer de mama, tumores sólidos em crianças e linfomas agressivos.^5^ No entanto, estudos sugerem que a cardiotoxicidade da DOX promova uma diminuição na fração de ejeção do ventrículo esquerdo (FEVE). A incidência de cardiomiopatias em pacientes tratados anteriormente ou em tratamento atual com DOX é de 3% a 26%, mas os dados sobre a prevalência ainda são escassos.^6^ A diminuição na FEVE pode iniciar com as primeiras doses de DOX e está relacionada à dose cumulativa. Doses abaixo de 550 mg/m² podem reduzir a probabilidade de cardiomiopatias.^2^ Doses mais altas podem causar danos permanentes ao miocárdio, caracterizados por apoptose dos miócitos, que resulta em fibrose e consequente perda de função cardíaca.^3^

O estresse oxidativo potencializado pela DOX parece dar início a uma série de processos bioquímicos nas fibras do músculo cardíaco, que resultam em danos ao retículo sarcoplasmático e à mitocôndria, modificações funcionais e estruturais das miofibrilas e modificação do acoplamento de excitação-contração e do fluxo de cálcio. Essas alterações levam à apoptose e, por fim, à perda da capacidade de regeneração do músculo cardíaco.^7^

Alguns dos benefícios do exercício são a melhora do funcionamento do sistema imunológico, a redução de atividade inflamatória e a atenuação dos efeitos metabólicos da imobilidade e da quimioterapia, que tornam o exercício uma excelente ferramenta não farmacológica para reduzir os efeitos tóxicos da DOX e ajudar a melhorar a qualidade de vida dos pacientes submetidos a tratamento.^8^^,^^9^ O potencial cardioprotetor do exercício contra a cardiotoxicidade parece estar ligado a diversos mecanismos moleculares, como produção antioxidante aumentada, regulação de sinalização pró-apoptótica, limitação da renovação de miócitos, modulação da atividade cardíaca da proteína quinase ativada por monofostato de adenosina (AMPK), regulação negativa da autofagia cardíaca, redução do acúmulo cardíaco de DOX, entre outros.^10^^–^^12^

Estudos sobre exercício e disfunção cardíaca causada por cardiotoxicidade de DOX em humanos ainda são limitados, mas há um número razoável de estudos pré-clínicos na literatura. Assim, esta metanálise foi realizada com estudos pré-clínicos e, até onde sabemos, é a primeira sobre o assunto. O objetivo foi verificar os efeitos de exercício aeróbio na função cardíaca de murinos expostos a DOX.

## Métodos

### Critérios de Inclusão

Foram incluídos ensaios clínicos randomizados (ECRs) com modelos de murinos que realizaram exercício aeróbio antes, durante e após a exposição à DOX, comparados com o grupo controle. A função cardíaca deveria ter sido medida pela fração de encurtamento (ΔD%) e pela pressão desenvolvida no ventrículo esquerdo (PDVE).

### Critérios de Exclusão

Foram excluídos estudos com delineamentos diferentes dos ECRs, estudos em que o grupo de exercício experimental usou medicamento concomitante, estudos com humanos ou estudos sem média e desvio padrão dos resultados relativos à ΔD% e PDVE.

### Busca

A busca foi conduzida em dezembro de 2017 nas bases de dados digitais MEDLINE, LILACS, CENTRAL Cochrane, PEDro, CINAHL, ScienceDirect, SPORTDiscus, Scopus e Web of Science. Os descritores cardiotoxicidade, câncer, doxorrubicina, exercício e todos os sinônimos presentes nas bases de dados Medical Subject Headings e Descriptors in Health Sciences foram utilizados na busca.

### Dados Extraídos dos Estudos

Os tipos de exercícios, os protocolos de treinamento, as dosagens de infusões de DOX, as formas de aplicação, os períodos de exposição ao fármaco, os resultados da ΔD% e da PDVE (mmHg) e o tamanho das amostras utilizadas foram extraídos dos estudos selecionados para revisão.

Para a análise da função cardíaca *in vivo*, foi considerada a ΔD% do ventrículo esquerdo medida por ecocardiografia e Doppler. A ΔD% é um dos principais parâmetros a serem monitorados em pacientes expostos a terapias cardiotóxicas, já que é uma medida indicativa da função sistólica do ventrículo esquerdo.^13^^,^^14^

Uma avaliação da PDVE através de um transdutor posicionado no ventrículo esquerdo (VE), baseada no modelo de isolamento cardíaco de Langendorff, é comum nesses estudos. Assim, a análise *ex vivo* também foi considerada para verificar a capacidade de contração do VE.^15^

### Análise da Qualidade Metodológica

Um avaliador independente analisou o risco de viés em cada estudo incluído na metanálise através da ferramenta da Cochrane Collaboration para determinar o risco de viés em ensaios clínicos randomizados (2005-2007), disponível para *download* em http://www.cochrane-handbook.org. Seguindo as recomendações do Cochrane Handbook for Systematic Reviews of Interventions,^16^ foi usada a abordagem GRADE através do GRADEproGDT, disponível em https://gradepro.org/, para analisar o nível de evidências para cada desfecho (ΔD% e PDVE).

### Análise Estatística

O programa Review Manager v. 5.3, com desfecho contínuo, método estatístico de variância inversa, análise por modelo de efeitos aleatórios, medida de efeito por diferença da média e intervalo de confiança (IC) de 95%, foi usado para os estudos, para a metanálise e para ordenar os estudos por peso. O nível de significância adotado foi de 5%.

## Resultados

**Figura 1:**
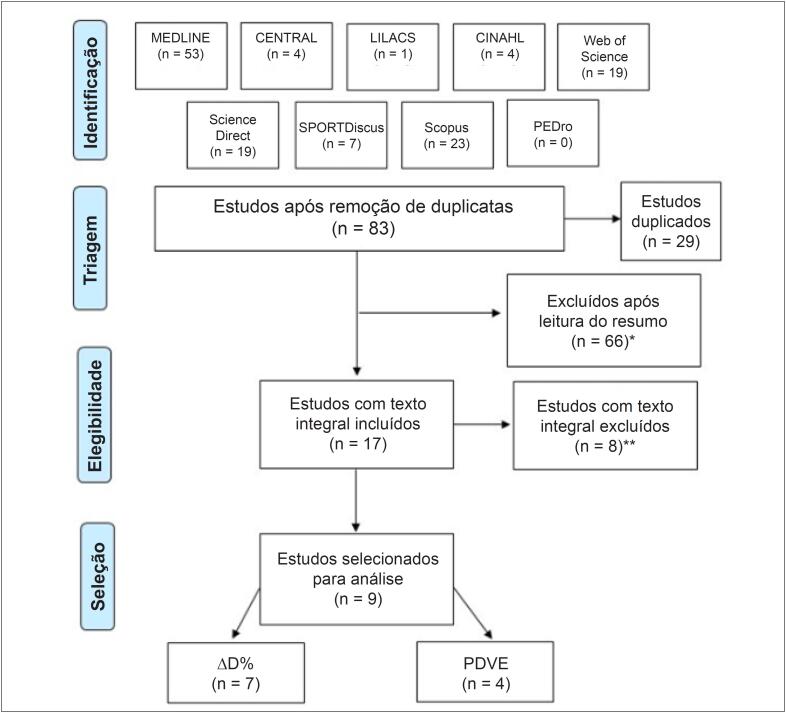
Diagrama de fluxo dos estudos (Prisma, 2009)18. *Estudos excluídos por não atenderem aos critérios de exclusão. **Estudos excluídos pelo uso concomitante de medicamento no grupo experimental, inclusão de seres humanos e/ou ausência de média e desvio padrão para a resposta da função cardíaca. ΔD%: fração de encurtamento; PDVE: pressão desenvolvida no ventrículo esquerdo.

As etapas realizadas na busca dos manuscritos estão descritas no diagrama da Figura 1. Entre os nove estudos selecionados para análise, sete apresentaram resultados de ΔD% e quatro apresentaram resultados de PDVE. Apenas dois estudos apresentaram as duas variáveis analisadas.

O estudo de Hydock et al.^19^ (2012) testou dois protocolos de injeção de DOX diferentes. Assim, foi dividido em duas análises, nomeadas “a” e “b”. Para verificar a influência do hormônio feminino na cardiotoxicidade induzida por DOX, o estudo de Calvé et al.,^20^ foi dividido em “a”, com ratos em condições normais, e “b”, com ratos ovariectomizados. Em uma análise com dois protocolos de exercício diferentes, o estudo de Jensen et al. (2013)^22^ foi dividido em “a”, em que os ratos foram submetidos ao protocolo de esteira progressiva, e “b”, em que o grupo tinha acesso livre à roda de corrida. Para entender melhor os resultados e as comparações, Lien et al.,^24^ (2015) conduziram quatro estudos (“a”, “b”, “c” e “d”), de acordo com o número de grupos ativos (Tabelas 1 e 2).

**Tabela 1:** Resumo dos estudos selecionados para a variável fração de encurtamento (ΔD%) do ventrículo esquerdo.

Autor (ano)	Tipo de exercício	Protocolo de intervenção	Injeção de DOX	Fração de encurtamento do ventrículo esquerdo (ΔD%)			
				Controle + DOX		Exercício aeróbio + DOX	
				n	${\bar{\text{X}}}_{\text{DP}}$	n	${\bar{\text{X}}}_{\text{DP}}$
Hayward et al. (2012)^18^	Aeróbio Roda de corrida	Acesso livre 24 h/dia Total: 10 semanas	2 mg/kg por 7 dias Total = 14 mg/kg durante o exercício	15	52 ± 38	17	61 ± 29
Hydock et al. (2012) _(a)_^19^	Aeróbio Roda de corrida	Acesso livre 24 h/dia Total: 10 semanas	1 mg/kg por 15 dias Total = 15 mg/kg durante o exercício	15	45 ± 3	9	46 ± 4
Hydock et al. (2012) _(b)_^19^	Aeróbio Roda de corrida	Acesso livre 24 h/dia Total: 10 semanas	2,5 mg/kg semanalmente por 6 semanas Total = 15 mg/kg durante o exercício	10	52 ± 5	10	61 ± 4
Calvé et al. (2012) _(a)_^20^	Aeróbio Nado	1 h/dia Total: 4 semanas	3 mg/kg no 26º dia de vida pré-exercício	8	53,1 ± 3,8	8	49,5 ± 2,2
Calvé et al. (2012) **(b)**^20^	Aeróbio Nado	1 h/dia Total: 4 semanas	3 mg/kg no 26º dia de vida pré-exercício	8	47 ± 2,1	8	51,6 ± 1,7
Dolisnky et al. (2013)^21^	Aeróbio Esteira	10-18 m/min 5 dias/semana Total: 8 semanas	8 mg/kg por semana por 4 semanas Total = 32 mg/kg pré-exercício	8	23,8 ± 1,0	8	28,0 ± 0,7
Jensen et al. (2013) _(a)_^22^	Aeróbio Esteira	13-30 min/m 5º-18º 20-60 min/dia 5 dias/semana Total: 10 semanas	10 mg/kg dose única pós-exercício	8	50,47 ± 2,77	4	61,60 ± 7,28
Jensen et al. (2013) _(b)_^22^	Aeróbio Roda de corrida	Acesso livre 24 h/dia Total: 10 semanas	10 mg/kg dose única pós-exercício	8	50,47 ± 2,77	7	58,3 ± 4,33
Parry et al. (2015)^23^	Aeróbio Roda de corrida	Acesso livre 24 h/dia Total: 11 semanas	12 mg/kg dose única pós-exercício	6	59 ± 6†	4	63 ± 4†
Lien et al. (2015) _(a)_^24^	Aeróbio Esteira	18-24 m/min Total: 5 dias	10 mg/kg dose única pós-exercício	10	48 ± 4	10	56 ± 4
Lien et al. (2015) _(b)_^24^	Aeróbio Roda de corrida	Acesso livre 24 h/dia Total: 5 dias	10 mg/kg dose única pós-exercício	10	48 ± 4	10	51 ± 5
Lien et al. (2015) _(c)_^24^	Aeróbio Esteira	18-24 m/min Total: 5 dias	15 mg/kg dose única pós-exercício	13	39 ± 6	13	48 ± 5
Lien et al. (2015) _(d)_^24^	Aeróbio Roda de corrida	Acesso livre 24 h/dia Total: 5 dias	15 mg/kg dose única pós-exercício	13	39 ± 6	12	45 ± 3

DOX: doxorrubicina; x: média; DP: desvio padrão;

† medidas feitas no 5º dia após a injeção de DOX;

(a), (b), (c), (d) são subdivisões dos estudos de Hydock et al.^19^ (2012), Calvé et al.^20^ (2012), Jensen et al.^22^ (2013) e Lien et al.^24^ (2015).

**Tabela 2:** Resumo dos estudos selecionados para a variável pressão desenvolvida no ventrículo esquerdo (PDVE), mmHg

Autor (ano)	Tipo de exercício	Protocolo de intervenção	Injeção de DOX	Pressão desenvolvida no ventrículo esquerdo (PDVE), mmHg			
				Controle + DOX		Exercício aeróbio + DOX	
				n	${\bar{\text{X}}}_{\text{DP}}$	n	${\bar{\text{X}}}_{\text{DP}}$
Chicco et al. (2005)^25^	Aeróbio Roda de corrida	Acesso livre 24 h/dia Total: 8 semanas	10-μM dose única pós-exercício	7	30,5 ± 1,4	7	50,1 ± 7,7
Chicco et al. (2006)^26^	Aeróbio Esteira	15-27 m/min 0º-5º 20-60 min/dia 5 dias/semana Total: 12 semanas	15 mg/kg dose única pós-exercício	15	46 ± 9	15	84 ± 7
Hayward et al. (2012)^18^	Aeróbio Roda de corrida	Acesso livre 24 h/dia Total: 10 semanas	2 mg/kg por 7 dias Total = 14 mg/kg durante o exercício	22	91 ± 15 †	22	121 ± 12 †
Jensen et al. (2013) _(a)_^22^	Aeróbio Esteira	13-30 min/m 5º-18 m 20-60 min/dia 5 dias/semana Total: 10 semanas	10 mg/kg dose única pós-exercício	14	70 ± 3††	10	93 ± 3††
Jensen et al. (2013) _(b)_^22^	Aeróbio Roda de corrida	Acesso livre 24 h/dia Total: 10 semanas	10 mg/kg dose única pós-exercício	14	70 ± 3††	10	89 ± 2††

DOX: doxorrubicina; x: média; DP: desvio padrão; (a) e (b) são subdivisões de Jensen et al.22 (2013);

† medida feita com 300 batimentos por minuto;

†† medida feita no 9º dia após a injeção de DOX.

**Figura 2:**
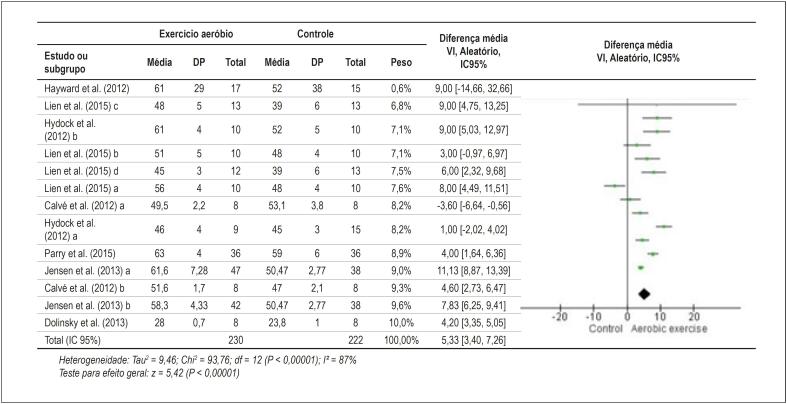
Gráfico de floresta dos estudos com murinos expostos à DOX que compararam a ΔD% de um grupo que realizou exercício aeróbio à de um grupo controle sedentário. DP: desvio padrão; DOX: doxorrubicina; ΔD%: fração de encurtamento; VI: variância inversa; IC: intervalo de confiança.

Os resultados da ΔD%, da PDVE e da I² desta metanálise são mostrados nas Figuras 2 e 3.

**Figura 3:**
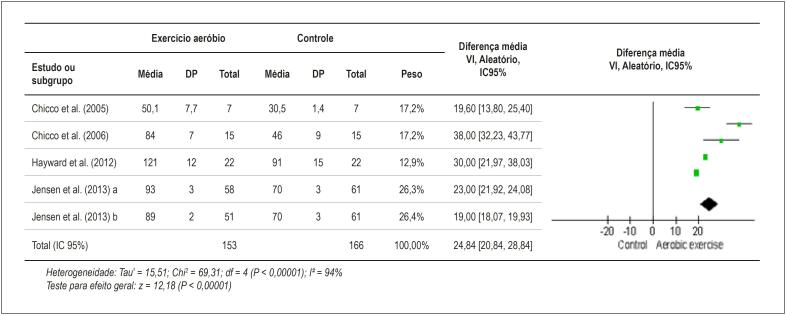
Gráfico de floresta dos estudos com murinos que compararam a PDVE de um grupo que realizou exercício aeróbio à de um grupo controle sedentário. DP: desvio padrão; PDVE: pressão desenvolvida no ventrículo esquerdo; VI: variância inversa; IC: intervalo de confiança.

O risco de viés dos estudos e o nível de evidência da metanálise são mostrados nas Tabelas 3 e 4, respectivamente.

**Tabela 3:** Ferramenta para avaliação de risco de viés da Cochrane Collaboration

Autor (ano)	Randomização	Ocultação da randomização	Cegamento dos participantes*	Cegamento dos avaliadores*	Desfechos incompletos	Relato seletivo de desfechos	Outras fontes de viés	Risco de viés
Chicco et al. (2005)^25^	Baixo	Baixo	Baixo	Baixo	Baixo	Baixo	Baixo	Baixo
Chicco et al. (2006)^26^	Baixo	Baixo	Baixo	Baixo	Baixo	Baixo	Baixo	Baixo
Hayward et al. (2012)	Baixo	Baixo	Baixo	Baixo	Baixo	Baixo	Baixo	Baixo
Hydock et al. (2012)^18^	Baixo	Baixo	Baixo	Baixo	Baixo	Baixo	Baixo	Baixo
Calvé et al. (2012)^20^	Baixo	Baixo	Baixo	Baixo	Baixo	Baixo	Baixo	Baixo
Jensen et al. (2013)^22^	Baixo	Baixo	Baixo	Baixo	Baixo	Baixo	Baixo	Baixo
Dolionsky et al. (2013)^21^	Baixo	Baixo	Baixo	Baixo	Baixo	Baixo	Baixo	Baixo
Parry et al. (2015)^23^	Baixo	Baixo	Baixo	Baixo	Baixo	Baixo	Baixo	Baixo
Lien et al. (2015)^24^	Baixo	Baixo	Baixo	Baixo	Baixo	Baixo	Baixo	Baixo

* Os itens referentes à randomização e ao cegamento da amostra foram considerados como tendo baixo risco de viés mesmo quando não estava explícito no ensaio clínico randomizado, já que estudos com modelos de murinos neutralizam esses vieses.

**Tabela 4:** Ferramenta GRADE para análise do nível de evidência.

Avaliação de certeza							Nº de pacientes		Efeito		Certeza	Importância
Nº de estudos	Delineamento do estudo	Risco de viés	Inconsistência	Evidência indireta	Imprecisão	Outras considerações	Exercício aeróbio	Controle	Relativo (IC95)	Absoluto (IC95%)		
**Fração de encurtamento (avaliada com ecocardiografia e Doppler)**												
13	ensaios randomizados	não grave	não grave	muito graveᵃ	não grave	nenhum	230	222	–	média **5,33% maior** (3,4 a 7,26)	⨁⨁◯◯ BAIXA	CRÍTICA
**Pressão desenvolvida no ventrículo esquerdo (avaliada com transdutor de pressão)**												
5	ensaios randomizados	não grave	não grave	muito graveᵃ	não grave	nenhum	153	166	–	média **24,84 mmHg maior** (20,84 a 28,84)	⨁⨁◯◯ BAIXA	CRÍTICA

IC: intervalo de confiança; a: estudos com animais são considerados como evidência indireta.

## Discussão

No presente estudo, a disfunção cardíaca resultante do uso de DOX foi avaliada através de ΔD% e PDVE (mmHg), que estão relacionadas à função sistólica do ventrículo esquerdo. A metanálise dos resultados de 230 murinos submetidos a exercício aeróbio mais DOX e de 222 controles (tratados somente com DOX) mostrou uma melhora na ΔD% de 5,33 (p = 0,00001) nos murinos que realizaram exercício aeróbio (Figura 2). Da mesma forma, a metanálise dos resultados de 153 murinos submetidos a exercício aeróbio mais DOX e dos 166 controles (tratados somente com DOX) mostrou um aumento na PDVE de 24,84 mmHg (p = 0,00001) nos murinos que realizaram exercício aeróbio (Figura 3). Resumidamente, a prática de exercício aeróbio contribuiu para a melhora da função sistólica, ou seja, para a diminuição da disfunção cardíaca causada pelo uso de DOX.

A toxicidade das antraciclinas causa disfunção grave em todos os tecidos musculares. No entanto, as células do músculo cardíaco parecem acumular maiores quantidades de DOX do que as células dos músculos liso e esquelético.^27^ Por isso, a detecção precoce dos fatores de risco cardiovascular, a monitorização cuidadosa dos parâmetros das funções diastólica e sistólica do ventrículo esquerdo e a medição da FEVE e da pressão de enchimento do ventrículo esquerdo devem ser realizadas periodicamente em pacientes submetidos a quimioterapia para evitar a perda permanente de função do músculo cardíaco devido à cardiotoxicidade.^28^

O exercício aeróbio parece promover a liberação dos antioxidantes, protegendo, assim, a fibra cardíaca de danos causados pela liberação excessiva de espécies reativas de oxigênio após a exposição à DOX.^29^^–^^32^ Esse efeito oxidativo antiestresse é percebido quando o exercício é realizado sistematicamente, antes ou depois da exposição ao fármaco.^33^ Embora as células tenham um sistema antioxidante endógeno, os cardiomiócitos têm uma capacidade de ativação desse sistema muito baixa quando comparados com células de outros tecidos.^34^^,^^35^ Assim, o exercício aeróbio tem se mostrado uma boa estratégia não farmacológica para combater a cardiotoxicidade.^36^

Em seres humanos, a prática regular de exercício aeróbio de três a quatro vezes por semana por 40 minutos, com atividades de intensidade moderada a extrema, parece ter efeito direto na prevenção de doenças cardiovasculares independentemente de outros fatores de riso, além de contribuir para taxas mais baixas de mortalidade cardíaca.^37^^,^^38^ Essa frequência de exercícios também afeta a produção de radicais livres, o que protege pacientes treinados dos efeitos crônicos gerados pelo estresse oxidativo de atividades físicas diárias.^39^

Um estudo metaepidemiológico mostrou que a intervenção com exercício aeróbio tem efeito similar ao de fármacos como betabloqueadores e inibidores de enzima de conversão da angiotensina nas taxas de mortalidade e prevenção secundária em pacientes com doenças coronárias, reabilitação de acidente vascular cerebral e tratamento de insuficiência cardíaca.^40^ Assim, é importante considerar o tratamento não farmacológico com exercícios para pacientes expostos a intervenções que acentuam o risco de doenças cardiovasculares, como a quimioterapia.

Outro aspecto positivo do exercício está relacionado à fadiga, a qual, além de ser um sintoma primário de muitos eventos cardíacos, é comum em pacientes expostos a quimioterapia. Puetz, Beasman e O'Connor^41^ concluíram, em uma metanálise, que programas de exercício para reabilitação cardíaca são associados à percepção de aumento de energia e diminuição de fadiga.^41^

Os resultados encontrados nesta metanálise a favor do grupo submetido a exercícios aeróbios mais DOX são fortalecidos pelos achados de revisões sistemáticas prévias sobre o mesmo assunto, que mostraram que a capacidade do exercício aeróbio de prevenir e combater a cardiotoxicidade gerada pela exposição à DOX parece estar bem estabelecida em estudos com animais.^42^^,^^43^ No entanto, os mecanismos desse efeito ainda não foram completamente elucidados.^44^^,^^45^

Em uma revisão sobre os efeitos de exercício físico na resposta cardiovascular de pacientes com câncer de mama, Sturgeon et al.^46^ revelaram uma falta de estudos focados em cardiotoxicidade em humanos. Eles apontaram que, apesar de poucos estudos pré-clínicos indicarem uma diminuição na frequência cardíaca em repouso e na pressão arterial em pacientes que praticam exercício aeróbio durante e após a quimioterapia, esses parâmetros não são suficientes para indicar boa função cardíaca. Kirkham et al.^47^ no entanto, em um estudo recente de prova de conceito com pacientes com câncer de mama, encontraram resultados favoráveis na avaliação da função sistólica do grupo que praticou apenas uma sessão de exercício aeróbio de corrida na esteira de intensidade vigorosa até 24 horas antes do tratamento com DOX.

## Limitações

Para ambos os desfechos, a inconsistência entre os estudos foi bastante alta, com I² = 87% (p = 0,00001) para ΔD% e I² = 94% (p = 0,00001) para PDVE (Figuras 2 e 3). Tal inconsistência pode estar relacionada à variação ampla nos tipos de exercícios, nos protocolos de intervenção e na dosagem de DOX utilizada (Tabelas 1 e 2). Por isso, a análise de efeitos aleatórios dos resultados foi escolhida. No entanto, o resultado final obtido não parece ter sido afetado por essa grande heterogeneidade. Por exemplo, dos quatro estudos com maior peso para ΔD%, dois realizaram exercício antes da DOX e dois após a DOX, os protocolos variaram de 4 a 10 semanas e a dose total de DOX variou de 3 mg/kg a 32 mg/kg. Assim, não é possível concluir quais foram os melhores protocolos.

Esses achados dos estudos pré-clínicos fornecem apenas evidência indireta em relação à prática clínica. Assim, a ferramenta GRADE teve diminuição de dois níveis no item evidência indireta, o que resultou em um baixo nível de evidência para as variáveis verificadas.

## Conclusão

Esta metanálise mostrou que, em estudos com murinos expostos à DOX, o exercício aeróbio antes, durante ou depois da exposição, realizado em uma sessão única ou por até 3 meses, é uma boa estratégia para a manutenção da função do ventrículo esquerdo. Os estudos pré-clínicos mostraram que, nesse estágio da pesquisa, o exercício é uma boa estratégia não farmacológica para preservar a função cardíaca contra danos causados pela cardiotoxicidade por DOX.
