# Supplementary material for: Exercício Aeróbio e Função Cardíaca de Murinos Expostos à Doxorrubicina: uma Metanálise
Source: Arq Bras Cardiol. 2020 Nov 1;115(5):885–93. [Article in Portuguese] doi: 10.36660/abc.20190260 (PMC8452221; doi:10.36660/abc.20190260)
Supplement: Translation

# Translation: Aerobic Exercise and Cardiac Function of Murines Exposed to Doxorubicin: a Meta-Analysis

**Authors:** Mariana Inocêncio Matos, Ercole da Cruz Rubini, Frederico de Oliveira Meirelles, Elirez Bezerra da Silva

**Affiliations:** 1 Rio de JaneiroRJ Brazil Universidade do Estado do Rio de Janeiro - Programa de Pós-Graduação em Ciências do Exercício e do Esporte, Rio de Janeiro, RJ - Brazil; 2 Rio de JaneiroRJ Brazil Universidade do Estado do Rio de Janeiro - Grupo de Pesquisa em Ciências do Exercício e da Saúde, Rio de Janeiro, RJ - Brazil; 3 Rio de JaneiroRJ Brazil Universidade Estácio de Sá, Rio de Janeiro, RJ – Brazil

**Keywords:** Muridae, Exercise, Anti-Bacterial Agents, Doxorubicin, Meta-Analysis

## Abstract

**Background:**

Cardiotoxicity may be a consequence of treatments with doxorubicin (DOX).

**Objectives:**

To investigate the effect of aerobic exercise on the prevention of cardiac dysfunction in murines exposed to DOX.

**Method:**

A comprehensive search was conducted in 9 databases in December 2017. Studies that evaluated the cardiac function of murines exposed to DOX were included. The significance level adopted was 5%.

**Results:**

In a comparison between 230 murines that underwent aerobic exercise plus DOX treatment and 222 control murines (DOX treatment only), fractional shortening showed an improvement of 5.33% in favor of the experimental group (p = 0.00001). Left ventricle developed pressure also showed an increase of 24.84 mm Hg in favor of the group of 153 murines that performed exercise in comparison to the control group of 166 murines (p = 0.00001).

**Conclusion:**

Preclinical studies included in this meta-analysis indicated that exercise is a good nonpharmacological strategy for preserving post-DOX cardiac function.

## Introduction

Chemotherapy exposes a new panorama in oncology, in which the survival of patients has increased along with their vulnerability to acquired cardiotoxicity in advanced treatments.^1^ The effects of the toxicity generated by the antineoplastic agents used in the treatment may manifest immediately, during their administration, or even years later.^2^^,^^3^ Among the organs affected, the heart deserves special attention because heart failure, often acquired after chemical treatment, has an equal or worse prognosis when compared to cancers in the liver, intestine, bladder, prostate, breast, and ovary. Therefore, such complications may interrupt the treatment and compromise the probability of a cure.^4^

Doxorubicin (DOX) is an efficient chemotherapeutic agent in the fight against breast cancer, solid tumors in children, and aggressive lymphomas.^5^ However, studies suggest that DOX cardiotoxicity promotes a decrease in left ventricular ejection fraction (LVEF). The incidence of cardiomyopathies in patients previously or currently treated with DOX is 3% to 26%, but data on the prevalence are still scarce.^6^ The decrease in LVEF may begin with the first doses of DOX and is related to the cumulative dose. Doses below 550 mg/m^2^ may reduce the possibility of cardiomyopathies.^2^ Higher doses may cause permanent damage to the myocardium, characterized by apoptosis of the myocytes, resulting in fibrosis and consequent loss of cardiac function.^3^

Oxidative stress potentiated by DOX seems to initiate a series of biochemical processes in cardiac muscle fibers, which result in injury to the sarcoplasmic reticulum and mitochondria, structural and functional modification of myofibrils, and modification of the excitation-contraction coupling and calcium flux. These changes lead to apoptosis and, ultimately, loss of the regeneration capacity of the cardiac muscle.^7^

Improved immune system function, reduced inflammatory activity, and attenuated metabolic effects of immobility and chemotherapy are some of the benefits of exercise, making it an efficient nonpharmacological tool that can reduce the toxic effects of DOX and help improve the quality of life of patients undergoing treatment.^8^^,^^9^ The cardioprotective potential of exercise against cardiotoxicity seems to be linked to several molecular mechanisms, such as increased antioxidant production, regulation of proapoptotic signaling, limitation of myocyte turnover, modulation of cardiac AMP-activated protein kinase (AMPK) activity, negative regulation of cardiac autophagy, reduction in myocardial accumulation of DOX, and others.^10^^–^^12^

Studies on exercise and cardiac dysfunction caused by DOX cardiotoxicity in humans are still limited, but there is a reasonable number of preclinical studies in the literature. Therefore, this meta-analysis included preclinical studies and, to the best of our knowledge, is the first on the subject. The aim was to investigate the effects of aerobic exercise on the cardiac function of murines exposed to DOX.

## Methods

### Inclusion Criteria

Randomized controlled trials (RCTs) of murines that performed aerobic exercise before, during, and after exposure to DOX compared to a control group were included. The cardiac function should have been measured by fractional shortening (FS%) and left ventricular developed pressure (LVDP).

### Exclusion Criteria

We excluded studies that used different designs from RCTs, that used concomitant medication in the experimental exercise group, that included humans, or that had no mean and standard deviation for FS% and LVDP results.

### Search

The search was conducted in December 2017 using the MEDLINE, LILACS, CENTRAL Cochrane, PEDro, CINAHL, ScienceDirect, SPORTDiscus, Scopus, and Web of Science electronic databases. The descriptors cardiotoxicity, cancer, doxorubicin, exercise, and all synonyms present in the Medical Subject Headings and Descriptors in Health Sciences databases were used in the search.

### Data Extracted from the Studies

The types of exercises, training protocols, dosages of DOX infusions, forms of administration, period of exposure to the drug, FS% and LVDP (mm Hg) results, and sample sizes were extracted from the studies selected for review.

For the *in vivo* analysis of cardiac function, left ventricular FS% measured by echocardiography and Doppler was considered. FS% is one of the main parameters to be monitored in patients exposed to cardiotoxic therapies, as this is an indicative measure of left ventricular systolic function.^13^^,^^14^

An evaluation of LVDP using a pressure transducer positioned in the left ventricle (LV), based on the Langendorff cardiac isolation model, is common in such studies. Therefore, the *ex vivo* analysis was also considered to examine the contractile capacity of the LV.^15^

### Analysis of Methodological Quality

An independent evaluator analyzed the risk of bias in each study included in the meta-analysis using the Cochrane Collaboration tool for assessing risk of bias in randomized trials (2005-2007), available for download at http://www.cochrane-handbook.org. Following the recommendations of the Cochrane Handbook for Systematic Reviews of Interventions,^16^ the GRADE approach via GRADEproGDT, available at https://gradepro.org/, was used to analyze the level of evidence for each outcome (FS% and LVDP).

### Statistical analysis

Review Manager v. 5.3, with a continuous outcome, statistical method of inverse variance, random-effects model analysis, measure of effect by mean difference, and 95% confidence interval (CI), was used for the studies and for the meta-analysis and ordering of studies by weight. The significance level adopted was 0.05.

## Results

The steps performed in the search of the manuscripts are described in the diagram in Figure 1. Among the 9 studies selected for analysis, 7 had FS% results and 4 had LVDP results. Only 2 studies had both variables analyzed.

**Figure 1:** Flow diagram of the studies (Prisma, 2009)18. *Studies excluded because they did not fulfill the inclusion criteria; **Studies excluded because of concomitant use of medication in the experimental group, inclusion of humans, and/or absence of mean and standard deviation response for cardiac function. FS%: fractional shortening; LVDP: left ventricular developed pressure.

The study conducted by Hydock et al.^19^ (2012) tested 2 different DOX injection protocols. Therefore, it was divided into two analyses named “a” and “b”. To assess the influence of the female hormone on DOX-induced cardiotoxicity, the study performed by Calvé et al.^20^ (2012) was divided into “a” with normal rats and “b” with ovariectomized rats. Using 2 different exercise protocols, the study conducted by Jensen et al.^22^ (2013) was divided into “a”, in which the rats underwent a progressive treadmill protocol, and “b”, in which the rats had free access to the running wheel. To better understand the results and their comparisons, Lien et al.^24^ (2015) conducted four studies (“a”, “b”, “c”, “d”) according to the number of active groups (Tables 1 and 2).

**Table 1:** Summary of studies selected for the variable left ventricular fractional shortening (FS%).

Author (year)	Type of exercise	Intervention protocol	DOX injection	Left ventricular fractional shortening (FS%)			
				Control + DOX		Aerobic exercise + DOX	
				n	${\bar{\text{X}}}_{\text{SD}}$	n	${\bar{\text{X}}}_{\text{SD}}$
Hayward et al. (2012)^18^	Aerobic Running wheel	Free access 24h/day Total: 10 weeks	2 mg/kg for 7 days Total = 14 mg/kg during exercise	15	52 ± 38	17	61 ± 29
Hydock et al. (2012) _(a)_^19^	Aerobic Running wheel	Free access 24h/day Total: 10 weeks	1 mg/kg for 15 days Total = 15 mg/kg during exercise	15	45 ± 3	9	46 ± 4
Hydock et al. (2012) _(b)_^19^	Aerobic Running wheel	Free access 24h/day Total: 10 weeks	2.5 mg/kg weekly for 6 weeks Total = 15 mg/kg during exercise	10	52 ± 5	10	61 ± 4
Calvé et al. (2012) _(a)_^20^	Aerobic Swimming	1h/day Total: 4 weeks	3 mg/kg on the 26th day of life pre-exercise	8	53.1 ± 3.8	8	49.5 ± 2.2
Calvé et al. (2012)_(b)_^20^	Aerobic Swimming	1h/day Total: 4 weeks	3 mg/kg on the 26th day of life pre-exercise	8	47 ± 2.1	8	51.6 ± 1.7
Dolinsky et al. (2013)^21^	Aerobic Treadmill	10-18 m/min 5 days/week Total: 8 weeks	8 mg/kg per week for 4 weeks Total = 32 mg/kg pre-exercise	8	23.8 ± 1.0	8	28.0 ± 0.7
Jensen et al. (2013) _(a)_^22^	Aerobic Treadmill	13-30 min/m 5⁰-18⁰ 20-60 min/day 5 days/week Total: 10 weeks	10 mg/kg single dose post exercise	8	50.47 ± 2.77	4	61.60 ± 7.28
Jensen et al. (2013) _(b)_^22^	Aerobic Running wheel	Free access 24h/day Total: 10 weeks	10 mg/kg single dose post exercise	8	50.47 ± 2.77	7	58.3 ± 4.33
Parry et al. (2015)^23^	Aerobic Running wheel	Free access 24h/day Total: 11 weeks	12 mg/kg single dose post exercise	6	59 ± 6†	4	63 ± 4†
Lien et al. (2015) _(a)_^24^	Aerobic Treadmill	18-24 m/min Total: 5 days	10 mg/kg single dose post exercise	10	48 ± 4	10	56 ± 4
Lien et al. (2015) _(b)_^24^	Aerobic Running wheel	Free access 24h/day Total: 5 days	10 mg/kg single dose post exercise	10	48 ± 4	10	51 ± 5
Lien et al. (2015) _(c)_^24^	Aerobic Treadmill	18-24 m/min Total: 5 days	15 mg/kg single dose post exercise	13	39 ± 6	13	48 ± 5
Lien et al. (2015) _(d)_^24^	Aerobic Running wheel	Free access 24h/day Total: 5 days	15 mg/kg single dose post exercise	13	39 ± 6	12	45 ± 3

DOX: doxorubicin; x: mean; SD: standard deviation;

† measurement done on the 5th day after DOX injection;

(a), (b), (c), (d) are subdivisions of the studies conducted by Hydock et al.^19^ (2012), Calvé et al.^20^ (2012), Jensen et al.^22^ (2013), and Lien et al.^24^ (2015).

**Table 2:** Summary of studies selected for the variable left ventricular developed pressure (LVDP), mm Hg

Author (year)	Type of exercise	Intervention protocol	DOX injection	Left ventricular developed pressure (LVDP), mm Hg			
				Control + DOX		Aerobic exercise + DOX	
				n	${\bar{\text{X}}}_{\text{SD}}$	n	${\bar{\text{X}}}_{\text{SD}}$
Chicco et al. (2005)^25^	Aerobic Running wheel	Free access 24 h/day Total: 8 weeks	10 μM single dose post exercise	7	30.5 ± 1.4	7	50.1 ± 7.7
Chicco et al. (2006)^26^	Aerobic Treadmill	15-27 m/min 0°-5° 20-60 min/day 5 days/week Total: 12 weeks	15 mg/kg single dose post exercise	15	46 ± 9	15	84 ± 7
Hayward et al. (2012)^18^	Aerobic Running wheel	Free access 24 h/day Total: 10 weeks	2 mg/kg for 7 days Total = 14 mg/kg during exercise	22	91 ± 15†	22	121 ± 12†
Jensen et al. (2013)_(a)_^22^	Aerobic Treadmill	13-30 min/m 5º-18 m 20-60 min/day 5 days/week Total: 10 weeks	10 mg/kg single dose post exercise	14	70 ± 3††	10	93 ± 3††
Jensen et al. (2013)_(b)_^22^	Aerobic Running wheel	Free access 24 h/day Total: 10 weeks	10 mg/kg single dose post exercise	14	70 ± 3††	10	89 ± 2††

DOX: doxorubicin; x: mean; SD: standard deviation; (a) and (b) are subdivisions of the study conducted by Jensen et al. (2013);

† measurement done with 300 beats per minute;

†† measurement done on the 9th day after DOX injection.

The results for FS% and LVDP as well as I^2^ in this meta-analysis are shown in Figures 2 and 3.

**Figure 2:** Forest plot of the studies of murines exposed to doxorubicin that compared fractional shortening in a group that performed aerobic exercise and a sedentary control group. SD: standard deviation; IV: inverse variance; CI: confidence interval.

**Figure 3:** Forest plot of the studies of murines that compared left ventricular developed pressure in a group that performed aerobic exercise and a sedentary control group. SD: standard deviation; IV: inverse variance; CI: confidence interval.

The risk of bias in the studies and the level of evidence in the meta-analysis are shown in Tables 3 and 4, respectively.

**Table 3:** Cochrane collaboration tool for evaluation of the risk of bias

Author (year)	Randomization	Concealment of randomization	Blinding of participants*	Blinding of evaluators*	Incomplete outcomes	Selective outcome reporting	Other sources of bias	Risk of bias
Chicco et al. (2005)	Low	Low	Low	Low	Low	Low	Low	Low
Chicco et al. (2006)	Low	Low	Low	Low	Low	Low	Low	Low
Hayward et al. (2012)	Low	Low	Low	Low	Low	Low	Low	Low
Hydock et al. (2012)	Low	Low	Low	Low	Low	Low	Low	Low
Calvé et al. (2012)	Low	Low	Low	Low	Low	Low	Low	Low
Jensen et al. (2013)	Low	Low	Low	Low	Low	Low	Low	Low
Dolionsky et al. (2013)^21^	Low	Low	Low	Low	Low	Low	Low	Low
Parry et al. (2015)^23^	Low	Low	Low	Low	Low	Low	Low	Low
Lien et al. (2015)^24^	Low	Low	Low	Low	Low	Low	Low	Low

* The items referring to randomization and blinding of the sample were considered as low risk of bias even when not stated in the randomized controlled trial, given that studies of murine models neutralize these biases.

**Table 4:** GRADE tool for analysis of the level of evidence.

Certainty assessment							No. of patients		Effect		Certainty	Importance
No. of studies	Study design	Risk of bias	Inconsistency	Indirectness	Imprecision	Other considerations	Aerobic exercise	Control	Relative (95% CI)	Absolute (95% CI)		
**Fractional shortening (assessed with echocardiography and Doppler)**												
13	randomized trials	not serious	not serious	very seriousᵃ	not serious	none	230	222	–	mean 5.33% higher (3.4 to 7.26)	⨁⨁◯◯ Low	Critical
**Left ventricular developed pressure (assessed with pressure transducer)**												
5	randomized trials	not serious	not serious	very seriousᵃ	not serious	none	153	166	–	mean 24.84 mm Hg higher (20.84 to 28.84)	⨁⨁◯◯ Low	Critical

CI: confidence interval; a: animal studies are considered indirect evidence.

## Discussion

In the present study, cardiac dysfunction resulting from DOX was evaluated using FS% and LVDP (mm Hg), which are related to left ventricular systolic function. A meta-analysis of the results of 230 murines that underwent aerobic exercise plus DOX treatment and 222 control murines (DOX treatment only) showed a FS% improvement of 5.33 (p = 0.00001) in those that performed aerobic exercise (Figure 2). Similarly, a meta-analysis of the results of 153 murines that underwent aerobic exercise plus DOX treatment and 166 control murines (DOX treatment only) showed a LVDP increase of 24.84 mm Hg (p = 0.00001) in those that performed aerobic exercise (Figure 3). In short, aerobic exercise contributed to improve the systolic function, i.e., to decrease the cardiac dysfunction caused by the use of DOX.

The toxicity of anthracyclines causes severe dysfunction in all muscle tissues. However, the cells in the cardiac muscle appear to accumulate higher amounts of DOX than the cells in the smooth and skeletal muscles.^27^ Thus, early detection of cardiovascular risk factors, careful monitoring of parameters of left ventricular systolic and diastolic function, and measurement of LVEF and left ventricular filling pressure should be performed periodically in patients undergoing chemotherapy in order to avoid permanent loss of cardiac muscle function due to cardiotoxicity.^28^

Aerobic exercise appears to promote the release of antioxidants, thus protecting the cardiac fiber from damage caused by excessive release of reactive oxygen species after exposure to DOX.^29^^–^^32^ This oxidative anti-stress effect is noticed when exercise is performed systematically, before or after exposure to the drug.^33^ Although the cells are endowed with an endogenous anti-oxidant system, cardiomyocytes have a very low capacity for activation of this system when compared with cells from other tissues.^34^^,^^35^ Thus, aerobic exercise has proved to be a good nonpharmacological strategy to fight cardiotoxicity.^36^

In humans, regular aerobic exercise 3 to 4 times a week for 40 minutes using moderate-to-extreme intensity activities seems to have a direct effect on the prevention of cardiovascular diseases, regardless of other risk factors, and contributes to lower the rates of cardiac mortality among those who practice it.^37^^,^^38^ This frequency of exercise also affects the production of free radicals, protecting trained patients from the chronic effects generated by the oxidative stress of daily physical activities.^39^

A metaepidemiological study showed that an aerobic exercise intervention had a similar effect to drugs such as beta-blockers and angiotensin-converting enzyme inhibitors on mortality rates and secondary prevention in patients with coronary diseases, stroke rehabilitation, and treatment of heart failure.^40^ Thus, it is important to consider the nonpharmacological treatment with exercise for patients exposed to interventions that accentuate the risk of cardiovascular diseases, such as chemotherapy.

Another positive aspect of exercise is related to fatigue, which, in addition to being a primary symptom of many cardiac events, is common in patients exposed to chemotherapy. Puetz, Beasman, and O'Connor^41^ concluded in a meta-analysis that exercise programs for cardiac rehabilitation are associated with the perception of increased energy and decreased fatigue.^41^

The results found in this meta-analysis in favor of the group that underwent aerobic exercise plus DOX are strengthened by the findings of previous systematic reviews on the subject, which showed that the ability of aerobic exercise to prevent and fight cardiotoxicity generated by DOX exposure appears to be well established in animal studies.^42^^,^^43^ However, the mechanisms of this effect have not yet been fully clarified.^44^^,^^45^

In a review of the effects of physical exercise on cardiovascular response in patients with breast cancer, Sturgeon et al.^46^ revealed a lack of studies focused on cardiotoxicity in humans. They showed that although a few preclinical studies indicate a decrease in resting heart rate and blood pressure in patients who practiced aerobic exercise during and after chemotherapy, these parameters are not sufficient to indicate good cardiac function. Kirkham et al.^47^ however, in a recent proof-of-concept study of patients with breast cancer, found favorable results when assessing the systolic function of the group that practiced only 1 aerobic exercise session of vigorous-intensity treadmill running up to 24 hours prior to DOX treatment.

### Limitations

For both outcomes, the inconsistency between the studies was very high, with I^2^ = 87% (p = 0.00001) for FS% and I^2^ = 94% (p = 0.00001) for LVDP (Figures 2 and 3). Such inconsistency may be related to the wide variation in types of exercises, intervention protocols, and DOX dosage used (Tables 1 and 2). Thus, a random-effects analysis of the results was chosen. However, the final result does not seem to have been affected by this great heterogeneity. For example, of the 4 studies with the highest weight for FS%, 2 performed exercise before DOX and 2 after DOX, protocols ranged from 4 weeks to 10 weeks, and total DOX dose ranged from 3 mg/kg to 32 mg/kg. Therefore, it was not possible to conclude which protocols were the best.

These findings from preclinical studies provide only indirect evidence regarding clinical practice. Therefore, the GRADE tool had 2 levels lowered in the indirectness item, which resulted in a low level of evidence for the study variables.

## Conclusion

This meta-analysis showed that, in studies of murines exposed to DOX, aerobic exercise before, during, or after exposure, performed in a single session or for up to 3 months, is a good strategy for maintenance of left ventricular function. Preclinical studies showed that, at this stage of research, exercise was a good nonpharmacological strategy to preserve cardiac function against damage caused by DOX cardiotoxicity.
